# The REPAIR study: oral antibiotics to prevent infection and wound dehiscence after obstetric perineal tear—a double-blinded placebo controlled randomized trial

**DOI:** 10.1186/s13063-024-08069-x

**Published:** 2024-03-27

**Authors:** Kathrine Perslev, Niels Klarskov, Thomas Bergholt, Hanna Jangö

**Affiliations:** 1 Department of Obstetrics and Gynecology, Herlev University Hospital, Borgmester Ib Juuls Vej 1, 15, Herlev, 2730 Denmark; 2 Department of Clinical Medicine, University of Copenhagen, Nørregade 10, Copenhagen, 1165 Denmark

**Keywords:** Obstetric tear, Women’s health, Antibiotics, Infection, Wound dehiscence, Randomized controlled trial

## Abstract

**Background:**

Approximately 85% of women experience an obstetric tear at delivery and up to 25% subsequently experience wound dehiscence and/or infection. Previous publications suggest that intravenous antibiotics administrated during delivery reduces this risk. We do not know if oral antibiotics given after delivery can reduce the risk of wound dehiscence or infection. Our aim is to investigate whether three doses of oral antibiotics (amoxicillin 500 mg/clavulanic acid 125 mg) given after delivery can reduce the risk of wound dehiscence and infection in patients with a second-degree obstetric tear or episiotomy.

**Methods:**

We will perform a randomized, controlled, double-blinded study including 221women in each arm with allocation 1:1 in relation to the randomization. The study is carried out at Department of Obstetrics & Gynecology, Herlev University Hospital, Copenhagen, Denmark. The women will be included after delivery if they have had a second-degree tear or episiotomy. After inclusion, the women will have a clinical follow-up visit after 1 week. The tear and healing will be evaluated regarding signs of infection and/or dehiscence. The women will again be invited for a 1-year clinical examination including ultrasound. Questionnaires exploring symptoms related to the obstetric tear and possible complications will be answered at both visits. Our primary outcome is wound dehiscence and/or wound infection, which will be calculated using *χ*^2^ tests to compare groups. Secondary outcomes are variables that relate to wound healing, as pain, use of painkillers and antibiotics, need for further follow-up, as well as outcomes that may be related to the birth or healing process, urinary or anal incontinence, symptoms of prolapse, female body image, and sexual problems.

**Discussion:**

Reducing the risk of wound dehiscence and/or infection would decrease the number of control visits, prevent the need for longer antibiotic treatment, and possibly also decrease both short-term and long-term symptoms. This would be of great importance so the mother, her partner, and the baby could establish and optimize their initial family relation.

**Trial registration:**

The conduction of this study is approved the 2/2–2023 with the EU-CT number: 2022–501930-49–00. ClinicalTrials.gov Identifier: NCT05830162.

**Supplementary Information:**

The online version contains supplementary material available at 10.1186/s13063-024-08069-x.

## Background

Approximately 85% of women experience an obstetric tear at delivery [1, 2]. A second-degree tear is the most common type, and it involves the skin, vagina, and the underling muscles of the pelvic floor. Up to 25% of women with this form of tear subsequently develop wound infection or dehiscence [1, 3]. Wound infection, defined as abscess formation or secretion of pus from the rupture [4], can cause painful and reduced healing and wound dehiscence as well as long-term consequences, for example dyspareunia and weakening of the pelvic floor. Wound dehiscence is defined as diastasis of more than 5 mm between the wound edges and is seen in up to 25% in women with a second-degree tear or an episiotomy [1, 3].

A recent large randomized controlled study found that intravenously administered antibiotics during instrumental delivery could reduce the risk of maternal infections in the maternity period [5]. In the study, the authors found as an incidental finding that women receiving antibiotics had a 50% reduced risk of obstetric tear wound dehiscence and infection. Another study also reported a lower prevalence of wound dehiscence and infection in the group of women that received antibiotic treatment during delivery, although the association between antibiotics and risk of complications after obstetric tear was not their primary outcome [3]. In Denmark, it is not the standard treatment to prescribe antibiotics to women with a second-degree tear or an episiotomy. We therefore want to investigate if orally administrated antibiotics within the first day after suture of a second-degree perineal tear or an episiotomy reduces the risk of wound dehiscence or infection.

## Methods

**Fig. 1:**
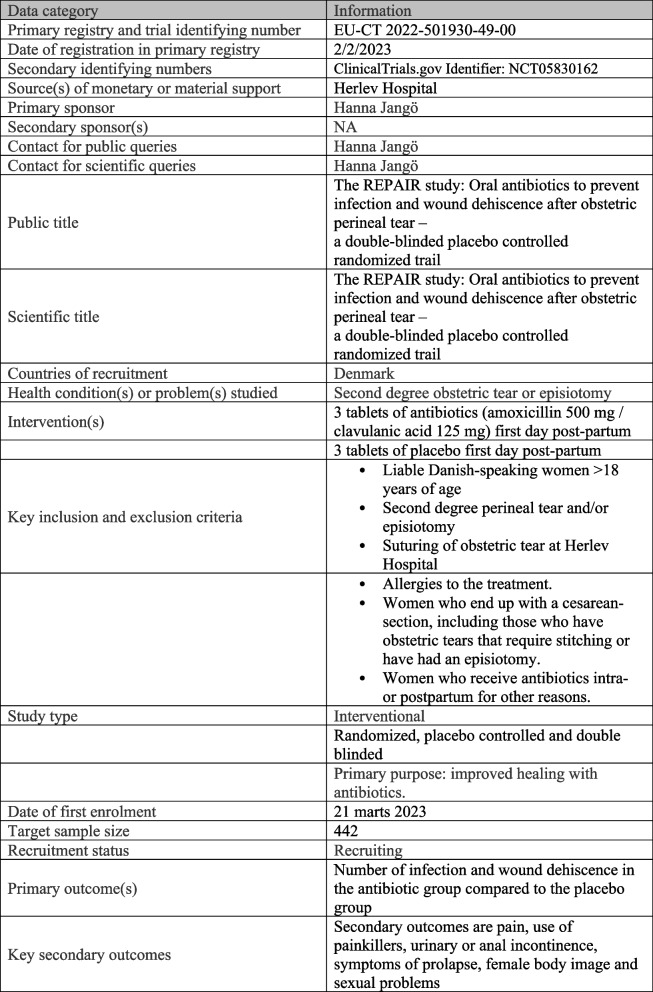
WHO trial registration data set

**Fig. 2:**
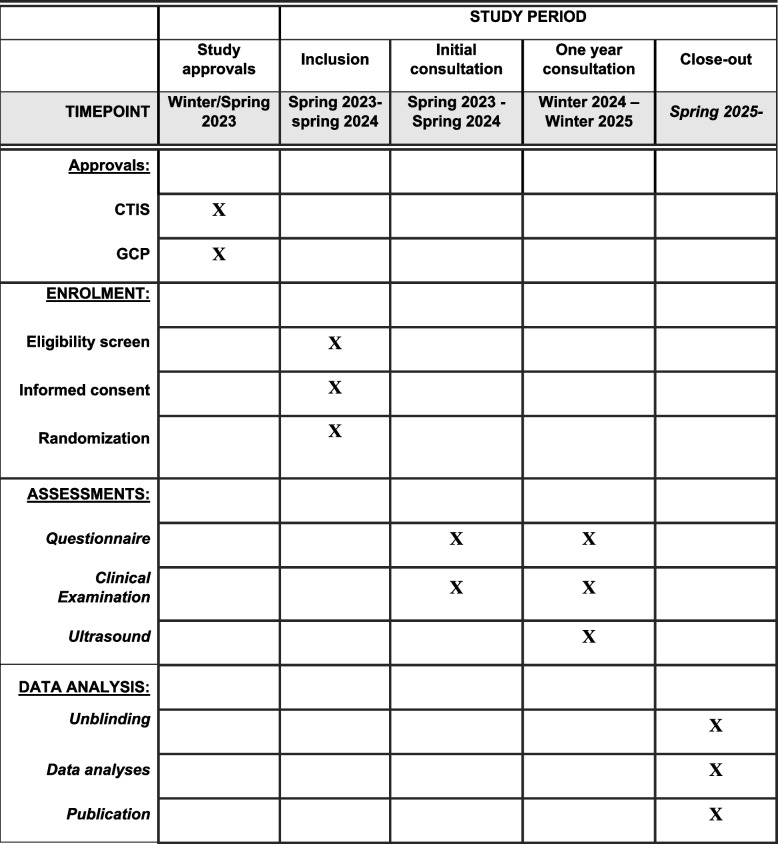
SPIRIT figure

The REPAIR study is a randomized, placebo-controlled, double-blinded trial investigating the effect of prophylactic antibiotic to women with a second-degree obstetric tear or episiotomy on risk of infection and/or wound dehiscence. The study will include 221 women in each arm with allocation 1:1 in relation to the randomization. The study is carried out at the Department of Obstetrics & Gynecology, Herlev University Hospital, Copenhagen, Denmark. We used the SPIRIT reporting guidelines in the protocol [6], and checklists are submitted (see Additional file 1). For additional trial registration information and expected timeline of this study, see Figs. 1 and 2.

## Study set up

### Information about the project

Participants should start the preventive treatment with antibiotics shortly after birth to have the intended effect, and therefore, the usual reflection time of 24 h cannot be given in this study. To optimize the decision process and support the women in making a well-informed confirmed consent, information about the project will be available in the antenatal period. Pregnant women at Herlev Hospital will receive information by digital post, by posters in the departments, as well as during birth preparation classes where the primary investigators will be present to give information and answer questions. Further contact information will be available for the women to use if they have any questions concerning the study. Thus, the women should be better prepared to give informed consent within a few hours after delivery.

The Danish monitor “Good Clinical Practice unit” (GCP) will monitor and approve the study before beginning of inclusion and during the entire study period.

### Screening

Physicians attending the labor ward are specially trained in the inclusion process. The primary investigators are responsible for this training. Physicians are hereafter responsible for screening all women with a second-degree tear or episiotomy for potential enrollment in the study based on the following inclusion and exclusion criteria.

Inclusion criteria.

- Liable Danish-speaking women > 18 years of age
- Second degree perineal tear and/or episiotomy
- Suturing of obstetric tear at Herlev Hospital

Exclusion criteria.

- Allergies to the treatment
- Women who end up with a cesarean-section, including those who have obstetric tears that require stitching or have had an episiotomy
- Women who receive antibiotics intra- or postpartum for other reasons

### Inclusion

Each potential candidate for inclusion in the study will be informed about the purpose and content of the project by a medical doctor in accordance with the recommendations of the Scientific Ethics Committee. The information covers the possible advantages and disadvantages and will be easy understandable. If a woman wants to participate in the trial, the consent form is signed.

All included women are informed that they can withdraw their consent to participate in the project at any time and without reason, without affecting current or later treatment. They are also given the leaflet “Your rights as a subject in a biomedical research project from the Central Scientific Ethics Committee.”

After inclusion, the physician including a woman in the study is responsible for handling the project medicine to her.

### Intervention

Placebo and amoxicillin 500 mg/clavulanic acid 125 mg will be obtained from The Capital Region Pharmacy, who will be responsible for the randomization process. The randomization will occur during the packing chronological ID-number medicine boxes each containing either placebo or the intervention medication. By establishing the randomization prior to enrollment, we avoid selection bias at the inclusion process. The medication will be stored in a locked medicine room at the maternity ward. The principial investigators will be responsible for control and handling of the trial medicine.

At inclusion, the women will be given the next chronological ID-number corresponding to a medicine box. The participant is then randomized to have either amoxicillin 500 mg/clavulanic acid 125 mg or placebo medicine for 1 day after delivery (three doses in total administrated with 8-h intervals). The blinding will be kept for both the participants, the including doctor, the principal investigators, and all four authors of this paper until the last visit for the last patient.

The randomization will be an individual level randomization with an allocation ratio of 1:1 to amoxicillin 500 mg/clavulanic acid 125 mg or placebo. Randomization will occur in blocks to reduce the risk of unblinding. The randomization process will not be stratified. The randomization will be based on a computer-generated random-number allocation sequence stored at the pharmacy. The randomization sequence will not be available to staff involved in data management, analysis, report writing, or patient care until breaking of the randomization code.

### Side effects and risks

Women are informed at inclusion about the medication, including possible side effects. The combination of amoxicillin and clavulanic acid is well-known and approved treatment and are given in a standard dose in a very short interval (three tablets over 24 h). The antibiotic can be used during breastfeeding [7], and given the combination of a short-term dosage before the milk production has started, we expect minimal side effects on the infants.

Side effects for the mother are also expected to be small due to the low dose and short-term treatment period. The assessment if a woman has experienced side effects to the medicine will be evaluated at the initial consultation after delivery. This cut off point for evaluation of side effect was chosen because amoxicillin with clavulanic acid has a half-life of 1 h, and it can therefore be expected that any side effects would have occurred at the first control visit 7–10 days after administration of the medicine. If the participant experience acute side effects after the first pill, she is informed not to continue the treatment but will still be able to continue for follow-up if desired. If a patient experience serious side effect and the doctor treating the patient wish to know if the participant had antibiotics, unblinding for that participant can be done. This unblinding will be handled by the investigator or sponsor by opening the code curve for that patient. The CGP-monitor will be informed about that directly hereafter.

### Initial consultation

The initial clinical consultation in the trial is scheduled 7–10 days after the delivery, with accepted intervals between 4 and 14 days after inclusion. The timepoint will be noted in the file to enable adjustment for this in the final analysis. In case of clinical symptoms before the initial consultation, the women are given detailed instructions to contact the Obstetric Department on a 24-h telephone line, thereby providing standard care.

At the initial consultation, the participant is asked to fill in a questionnaire regarding pain, urinary incontinence, and anal incontinence. Furthermore, she will be asked if she has been prescribed antibiotics for other reason since delivery, if she took all three doses of the study medication (compliance), and if she suffered from any medical side effects. All potential side effects will be evaluated by the physician performing the clinical examination. The tear will be examined by physicians specially trained for the study. In case of doubt about wound dehiscence or infection, other physicians will examine the participant to reach consensus.

In case of cancelation of the initial consultation, the participants will receive a personal phone call to clarify if she is willing to fill in the questionnaire and if she wants to be contacted for the 1-year clinical examination.

### One-year follow-up consultation

At the 1-year consultation, the participant fills in questionnaires about urinary incontinence, anal incontinence, prolapse problems, body image, breastfeeding, and sexual problems. At this visit, a gynecological and ultrasound examination will be carried out to assess the gynecological anatomical outcome after the birth and obstetric tear. We will evaluate the perineum, the pelvic floor muscles, the anal sphincter, and signs of genital prolapse. Ultrasound will be used to evaluate clinically undiagnosed obstetric anal sphincter injuries, possible defects of the perineal muscles, and damage to the levator ani.

### Outcome

The primary outcome of the trial is the difference in frequency of wound dehiscence and infection in the two groups at 7 to 10 days after delivery. Secondary outcomes are pain, use of painkillers and antibiotics prescribed post-partum, or the need for further follow-up. This information will be extracted from the questionnaire at the initial consultation. Other secondary outcomes are urinary or anal incontinence, symptoms of prolapse, female body image, and sexual problems. These long-term outcomes will be extracted from the questionnaire and the clinical examination at the 1-year consultation. The ultrasonically assessed substance of the perineal muscles, the levator ani muscle, and the anal sphincter are additional secondary outcomes.

### Data

All data are collected and stored confidentially in accordance with General Data Protection Regulation (GDPR). Data are stored in a separate approved and secure database (REDCap), which only the investigators and sponsor have access to, and only the investigators will enter data to reduce the risk of mistakes. Data are processed in Excel and the statistical software R and are stored in accordance with the Danish Capital Region’s requirements for research data. The GCP monitor will control the data entry by comparing REDCap information with the patient’s journal for 10% of the participants. The Danish GCP unit (https://gcp-enhed.dk/) is independent from the sponsor and competing interests and will audit the trial conduct with regular visits.

## Statistics

### Sample size calculation

Based on the study by Knight et al. [5], 6.5% in the antibiotic group had a subsequent superficial or deep infection after obstetric tear. The corresponding number for the placebo group was 12.8%. Similarly, they found a lower prevalence of wound dehiscence, 11% in the antibiotic treated group compared to 21% in the placebo group. Based on a Danish Cohort study, the risk of wound dehiscence in women with second degree perineal tears was 18% and the risk of wound infection 9% [3].

We assumed that the frequency of wound dehiscence and/or infection for second degree tear after vaginal delivery is approximately 20%. We further hypothesized that the risk of subsequent infection and/or dehiscence can be reduced by 50% by administering oral amoxicillin 500 mg/clavulanic acid 125 mg given as three doses in the first day after birth compared to placebo. With a dropout rate of 10%, 221 women must be included in each group, to find a significant difference between groups with a significance level of 0.05 and a power of 80%. The total number of women with second degree perineal tear to be included in this study is 442 with 1:1 randomization.

### Interim analysis

We plan to conduct an interim analysis when 50% of the planned participants are included. The interim analysis will be performed to ensure the expected frequency of wound dehiscence and/or infection in the over-all study population. The blinding of the study will be kept during the interim analysis. Since we expect a frequency of approximately 20% in the control group and approximately 10% in the antibiotic group, we would expect a frequency of 15% in the total trial population. If the interim analysis calculates a total frequency of approximately 15% or more, the study will be continued until the planned 442 women are included. If the interim analysis demonstrates a significantly lower total frequency than expected, we will calculate the additional number of women needed to be included to maintain the power in the study and extend the study period accordingly. The re-calculation will be based on the calculated frequency during the first half of the trial using the same expected effect of the intervention in reduction of wound dehiscence and/or infection.

### Outcome analysis

The unblinding of the data in the study takes place after the last participant has completed the 1-year control visit. The groups will be analyzed descriptively with median, range, and IQR (interquartile range) and quartiles 1 and 3 (Q1 and Q3). The results are calculated with proportion and percentage and with univariable and multivariable analyses. Our primary outcome is wound dehiscence and/or infection, which will be calculated using *χ*^2^ tests to compare the antibiotic group with the placebo group. Secondary outcomes related to long-term symptoms will be analyzed with *χ*^2^ test or Fisher’s exact test or with *t*-test or Mann–Whitney test depending on whether the data are normally distributed or not. All results will be analyzed based on intention-to-treat analysis. Missing data will be indicated for all variables and will basically be handled with case-wise deletion, where we leave the subject’s results omitted only for the analyses where data are missing. We will perform subanalyses focusing on wound dehiscence as clinically significant or insignificant as well as for wound dehiscence or infection that causes treatment or further follow-up visits. Statistical analyses are performed with the statistical software R (https://cran.r-project.org/).

### Publication

All results—regardless of the magnitude or direction of effect—will be published in peer-reviewed international papers and will be presented at national and international conferences. There are no publication restrictions for the trial. Authorships will be based on the criteria stated in the recommendations for publication from International Committee of Medical Journal Editors. All study participants will be offered information about the result of the study.

## Discussion

This randomized controlled double-blinded study evaluates whether oral antibiotics can prevent wound dehiscence or infection after second degree obstetric tears or episiotomy compared to placebo. The study has the possible advantage for the group receiving antibiotics that they might have a lower risk of infection and wound dehiscence compared to the control group. Conversely, this group also has a risk of side effects to the medication. The antibiotic used in this study is a frequently used medication that can also be used in breastfeeding women [7]. The placebo group will most likely not experience any side effects but conversely do not have the possible protective effect with respect to risk of infection or wound dehiscence. It is not standard in Denmark to treat second-degree tear or episiotomy with antibiotics and therefore the placebo group in this study is treated in accordance with national guidelines. Both groups are offered consultation with a physician in the first 2 weeks as well as 9–12 months after birth, which is not offered routinely today. This may be of concern to some of the women who will think more about the tear and recovery than they otherwise would have done. However, it can also have a calming effect on some of the trial participants that the tear and healing is being monitored and that it is assessed by a health care professional. For participants experiencing infection and/or wound dehiscence, the initial consultation can result in earlier diagnosis and treatment and, thus, potentially improved outcome. Conversely, some women might have tendency to postpone contact with their own general practitioner for a check-up, because they are waiting for the scheduled appointment in study. However, the participants are thoroughly informed about the importance of contacting a physician if they experience any discomfort before the initial consultation.

If our study finds a significant reduction in wound dehiscence and/or infection, introducing preventive oral antibiotic as a standard treatment for women with a second-degree tear or episiotomy, a frequent outcome in the vaginal delivery, needs to be considered thoroughly, especially since this population consist of breastfeeding women and due to the overall overconsumption of antibiotics worldwide. Antibiotics to breastfeeding women always have the potential to be transmitted to the baby with possible short- and long-term complications. In this study, the antibiotics are only administrated within the first 24 h of the delivery before the milk production has started. We therefore expect no clinically relevant doses transmitted to the baby. Notably, if the prophylactic antibiotics decreases the number of infections at the initial consultation, this study has the potential to reduce the amount of antibiotic prescribed at later timepoints where the risk of transmission to the baby is increased. With regard to the overall overconsumption of antibiotics, Knight et al. [5] found that the total use of antibiotics in the post-partum period was decreased for the participants who received prophylactic antibiotics compared to placebo. If this study support this finding, it has the potential to reduce the overall use of antibiotic in the postnatal period.

If this study finds that three doses of prophylactic antibiotics in women with a second-degree obstetric tear reduces the risk of infection and/or wound dehiscence by 50%, it will reduce the number of women with infections and/or wound dehiscence with 1500 annually in Denmark. The Number Needed to Treat (NNT) would be 10 to avoid one woman with infection or wound dehiscence. Optimizing the maternity period is essential for the mother, the child, and family formation, and it is therefore crucial to find ways to reduce the number of unnecessary stress factors, such as infections and wound dehiscence.

It is well-known that vaginal delivery and obstetric tears can cause long-term consequences like urinary and anal incontinence, pelvic organ prolapse, and affected sexual life, symptoms that can affect quality of life severely. Some studies indicate that second-degree tear can cause dyspareunia [8], decreased sexual activity 12 months postpartum [9], and increased perineal pain postpartum [10] compared to women with no perineal tear. However, it is unknown whether women with impaired healing with wound dehiscence or infection have a further increased risk of long-term complications. The results from our study can answer this question and show if prophylactic antibiotics decreases the risk of long-term symptoms.

## Trial status

This study is approved on 2 February 2023 with the EU-CT number: 2022–501930-49–00. Recruitment began on 21 March 2023. ClinicalTrials.gov Identifier NCT05830162. We expect the recruitment to be completed spring 2024.

### Supplementary Information

**Additional file 1. **SPIRIT Checklist.

**Additional file 2. **Ethical approval document.

**Additional file 3. **Copy of the original funding documentation.

## Data Availability

The four authors of this paper will have access to the final dataset when the last patient have had her last visit and the unblinding can be done.

## Abbreviations

GCP-unit: Good Clinical Practice unit
GDPR: General Data Protection Regulation
IQR: Interquartile range
NNT: Number Needed to Treat
