# Supplementary material for: The REPAIR study: oral antibiotics to prevent infection and wound dehiscence after obstetric perineal tear—a double-blinded placebo controlled randomized trial
Source: Trials. 2024 Mar 27;25:221. doi: 10.1186/s13063-024-08069-x (PMC10967187; doi:10.1186/s13063-024-08069-x)
Supplement: Supplementary file 2 — Additional file 2. Ethical approval document. [file 13063_2024_8069_MOESM2_ESM.pdf]

EUROPEAN MEDICINES AGENCY
SCIENCE MEDICINES HEALTH

Report for the Application Evaluation Decision

Oral antibiotics to prevent infection and wound dehiscence after obstetric perineal tear - a double-blinded placebo controlled randomized trail

2022-501930-49-00

Decision

MSC:

Denmark

Decision:

Authorised

Reporting Date:

02/02/2023

Tacit decision:

No

Application Part:

Conditions:

Reason:

Justification:

Deferrals

Disagreement with Part I

Submission date:

02/02/2023
