# Supplementary material for: The REPAIR study: oral antibiotics to prevent infection and wound dehiscence after obstetric perineal tear—a double-blinded placebo controlled randomized trial
Source: Trials. 2024 Mar 27;25:221. doi: 10.1186/s13063-024-08069-x (PMC10967187; doi:10.1186/s13063-024-08069-x)
Supplement: Supplementary file 3 — Additional file 3. Copy of the original funding documentation. [file 13063_2024_8069_MOESM3_ESM.pdf]

Enrolment certificate for kathrine Perslev

Name: kathrine Perslev
E-mail: kathrine.perslev@regionh.dk
Phone no.: 51235915
Date of birth: 31 August 1989

Start of study: 1 May 2023
End of study: 30 April 2026

Graduate programme: Surgical Sciences
Department: Department of Clinical Medicine

The project is carried out at: Herlev Hospital. Department of Gynaecology and Obstetrics
Place of employment: Herlev Hospital. Department of Gynaecology and Obstetrics

Project title: Oral antibiotics to prevent infection and wound dehiscence after obstetric perineal tear - a double-blinded placebo controlled randomized trail

Principal supervisor
Clinical Associate Professor Hanna Jangö
Department of Clinical Medicine
hanna.jango@regionh.dk

Primary co-supervisor
Clinical Professor Niels Klarskov
Herlev Hospital. Department of Gynaecology and Obstetrics
niels.klarskov@regionh.dk

Other supervisors
Professor Thomas Bergholt
Herlev Hospital. Department of Gynaecology and Obstetrics
thomas.bergholt@regionh.dk

External assessor

Submission of regular assessments
Regular assessments must be submitted as follows:

First assessment: 26 months before submission of PhD thesis
Second assessment: 14 months before submission of PhD thesis
Third assessment: 6 months before submission of PhD thesis

Please note that the submission dates will change accordingly if the PhD programme is extended.

Financial conditions
The tuition fee is guaranteed by **Jeannet Kepp Bruun Laueborg, Herlev Hospital** and must be paid to the Graduate School of Health and Medical Sciences, University of Copenhagen, in three instalments:

First rate:	DKK 50,000 is paid at enrolment
Second rate:	DKK 50,000 is paid in the first quarter of the calendar year following enrolment
Third rate:	DKK 50,000 is paid in the first quarter of the second calendar year following enrolment
